# Genovar: a detection and visualization tool for genomic variants

**DOI:** 10.1186/1471-2105-13-S7-S12

**Published:** 2012-05-08

**Authors:** Kwang Su Jung, Sanghoon Moon, Young Jin Kim, Bong-Jo Kim, Kiejung Park

**Affiliations:** 1 Division of Bio-Medical Informatics, Center for Genome Science, Korea National Institute of Health, Osong, 363-951, Korea; 2 Division of Structural and Functional Genomics, Center for Genome Science, Korea National Institute of Health, Osong, 363-951, Korea

## Abstract

**Background:**

Along with single nucleotide polymorphisms (SNPs), copy number variation (CNV) is considered an important source of genetic variation associated with disease susceptibility. Despite the importance of CNV, the tools currently available for its analysis often produce false positive results due to limitations such as low resolution of array platforms, platform specificity, and the type of CNV. To resolve this problem, spurious signals must be separated from true signals by visual inspection. None of the previously reported CNV analysis tools support this function and the simultaneous visualization of comparative genomic hybridization arrays (aCGH) and sequence alignment. The purpose of the present study was to develop a useful program for the efficient detection and visualization of CNV regions that enables the manual exclusion of erroneous signals.

**Results:**

A JAVA-based stand-alone program called Genovar was developed. To ascertain whether a detected CNV region is a novel variant, Genovar compares the detected CNV regions with previously reported CNV regions using the Database of Genomic Variants (DGV, http://projects.tcag.ca/variation) and the Single Nucleotide Polymorphism Database (dbSNP). The current version of Genovar is capable of visualizing genomic data from sources such as the aCGH data file and sequence alignment format files.

**Conclusions:**

Genovar is freely accessible and provides a user-friendly graphic user interface (GUI) to facilitate the detection of CNV regions. The program also provides comprehensive information to help in the elimination of spurious signals by visual inspection, making Genovar a valuable tool for reducing false positive CNV results. Availability: http://genovar.sourceforge.net/.

## Background

Recent advances in microarray and sequencing technology have enabled the identification of genomic variation in humans. Among the different types of variants, Single Nucleotide Polymorphisms (SNPs) and Copy Number Variation (CNV) have attracted attention due to the relatively frequency of their incidence in the human genome [1,2]. Genome-Wide Association Studies (GWAS) based on millions of SNP markers have been successful in discovering common variants responsible for the variation of complex phenotypes [3]. Given the complex traits associated with genetic variants, however, only a small fraction of heritability could be explained by established associations [3,4]. CNV is one of the potential alternative sources of the missing heritability components. Large, rare deletions within gene regions have been reported to be the causal loci for multiple complex phenotypes [5,6], while only a few common and small CNVs were identified as the associated loci [7].

The study of small CNVs may have been limited by the shortcomings of previously used platforms such as SNP genotyping and Comparative Genomic Hybridization array (aCGH) [7]. Although Next Generation Sequencing (NGS) technologies allowed the discovery of small CNVs, the currently available analytical tools are often associated with a high rate of false positive results [8]. The accuracy of CNV detection could be improved by visual inspection of the aligned reads. However, to the best of our knowledge, the currently available softwares such as CHESS, ISACGH, VAMP, and SIGMA2 do not support the visualization of aCGH and NGS data simultaneously [9-22]. Table 1 describes the feature comparison of CNV analysis softwares.

**Table 1:** Comparison of Genovar with previously reported CNV analysis tools

Plat- form	Module	CGHPRO	VAMP	ISACGH	MD-SeeGH	SIGMA2	CGcgh	CGHweb	CHESS	SnoopCGH	CNA-Reporter	SEURAT	Wavi-CGH	FISH Oracle	CNVAS	GENOVAR
	**Year**	**2005**	**2006**	**2007**	**2008**	**2008**	**2008**	**2008**	**2009**	**2009**	**2010**	**2010**	**2010**	**2011**	**2011**	**2011**
	
	CNV detection	O		O	O	O	O	O	O	O	O	O	O		O	O
CGH	CNV visualization	O	O	O	O	O	O	O	O	O	O	O	O	O	O	O
	CNV quality control	O														O
	Annotation	O		O	O	O	O	O	O	O	O	O	O	O	O	O

	CNV detection															O
NGS	CNV visualization															O
	CNV quality control															O
	Annotation															

The present study describes the development of a JAVA-based stand-alone program called Genovar that efficiently detects CNV regions and provides a visual inspection function to reduce false positive CNV calls based on aCGH and NGS data. Genovar can analyze both aCGH and NGS data (Table 1).

Genovar consists of three major components. Firstly, Genovar visualizes aCGH data and sequence alignment of chromosomal regions. Regarding NGS data, Genovar provides a read-depth plot, and summary information of each read when a certain read is selected in the panel. With respect to CNVs, the SW-array algorithm [23] was implemented for fast and easy CNV detection. Quality control assessment is the second functional component that filters out spurious signals by manual inspection on log2ratio signals, break points of aCGH data, read depth, and read alignment of NGS data. The last component provides highly reliable annotation derived from Database of Genomic Variants (DGV) [24] and Single Nucleotide Polymorphism Database (dbSNP) [25]. This is particularly useful to detect novel copy number aberrations. Since Genovar is capable of visualizing CNV data from variable sources such as aCGH data file and sequence alignments files from NGS studies, Genovar could be a useful software to visualize the CNVs with aCGH and NGS data. The feature for filtering out of spurious detections based on quality metrics enables to analyse CNV more conveniently and accurately via its useful functions.

## Results and discussion

### System architecture and features

**Figure 1:**
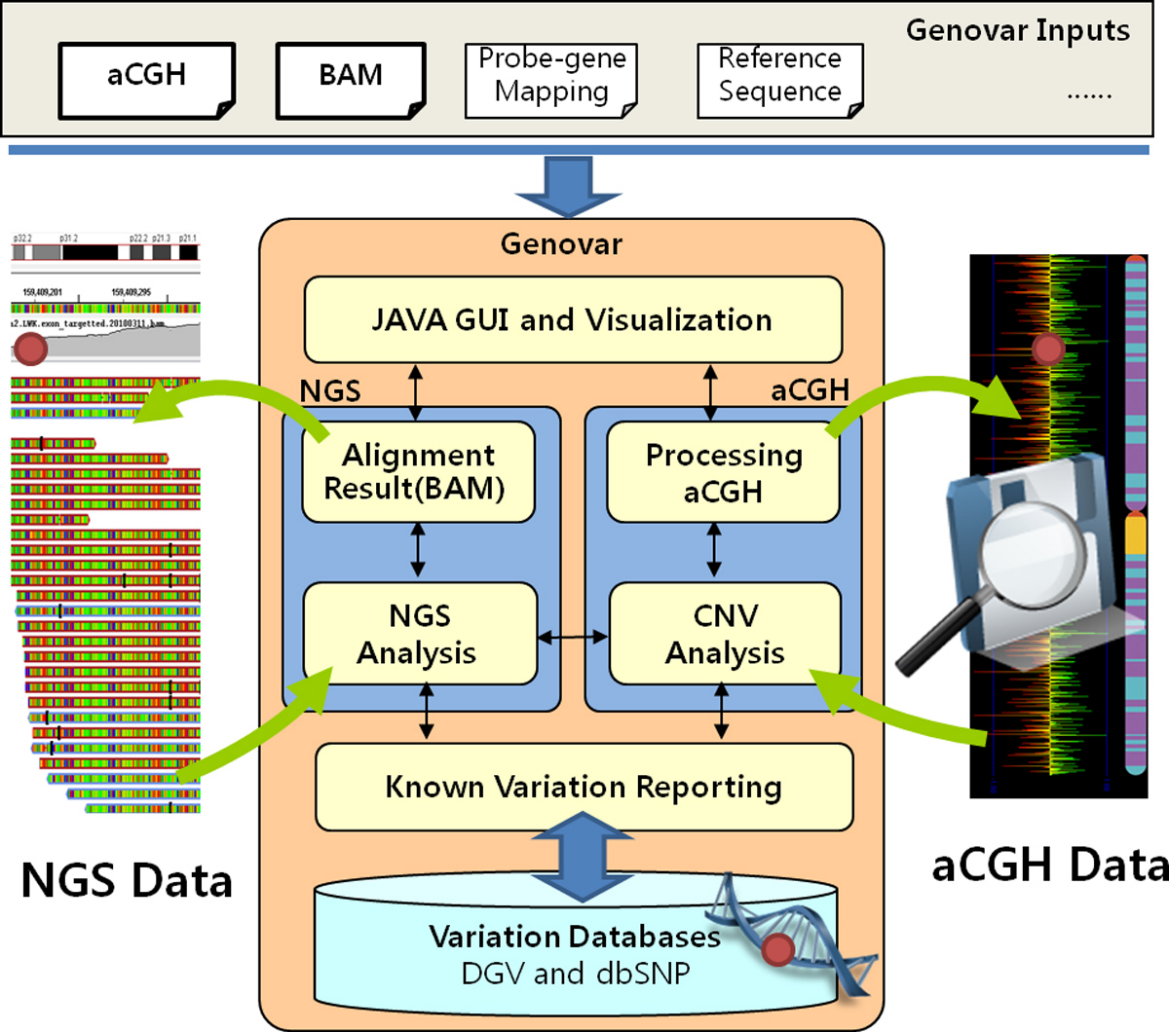
**System architecture of Genovar**. Genovar mainly takes comparative genomic hybridization array (aCGH) and binary sequence alignment/map (BAM) file as mandatory inputs for copy number variation (CNV) and next generation sequencing (NGS) analysis. Optional inputs include probe-gene mapping information and reference sequence (UCSC fastA format). Using two major modules (aCGH and NGS), Genovar performs analyzing tasks such as known variation reporting, graphical visual inspecting and variation detecting.

Genovar is a stand-alone application for the identification and visualization of CNV regions based on two major types of data input such as aCGH and Binary Alignment/Map (BAM) file formats. The graphical user interface was implemented by JAVA swing, and user interactions are handled by this intuitive interface. The Smith-Waterman Array (SW-ARRAY) algorithm [23] has been embedded into Genovar, and this algorithm provides a dynamic programming solution for detecting CNV regions. Because SW-ARRAY algorithm depends on a single threshold parameter, the results are more sensitive to changes of the threshold [23]. Figure 1 shows the system architecture of Genovar. Functions are widely categorized into two major modules, the analysis of aCGH data and the analysis of sequence alignment results. The features of the aCGH module are summarized below. For more detailed information and examples in terms of operating Genovar, a user's guide is given in Additional file 1.

• Analyzing/comparing them with multiple aCGH samples and windows.

• Elimination of spurious signals, and statistical block operation of log2 ratio values.

• Identification of CNV regions using the SW-Array algorithm and thresholds.

• Notation of known CNVs in comparison to newly identified CNV regions using DGV and dbSNP.

• Graphic representation of CNV region and aCGH information with multiple samples.

The features of the NGS module are as follows.

• User-intuitive and fast navigation on chromosomes for retrieving reads in terms of locus range query.

• Graphic-based display of sequence alignment results and read information in BAM (binary sequence alignment/map) format.

• Calculation of read-depth and allele frequency of each locus in the alignment area specified by the user and identification of known SNPs and CNV from dbSNP and DGV, respectively.

• Comparison of sequence alignment results between different samples.

### System inputs

As mentioned earlier, Genovar uses two major input data formats such as aCGH or NGS. First, the aCGH input format includes probe ID and name, chromosome number, probe starting and ending locus, and a series of log2 intensity values delimited by 'TAB'. The aCGH sample formats are available on the Genovar website http://genovar.sourceforge.net/. Second, Genovar uses a BAM format to perform sequence-based variation analysis. To display reference sequence (hg19/hg18) information, Genovar uses the UCSC FastA reference sequence format. Users can easily download FastA formats from the UCSC website http://genome.ucsc.edu/.

### Chromosomal view from array CGH data

**Figure 2:**
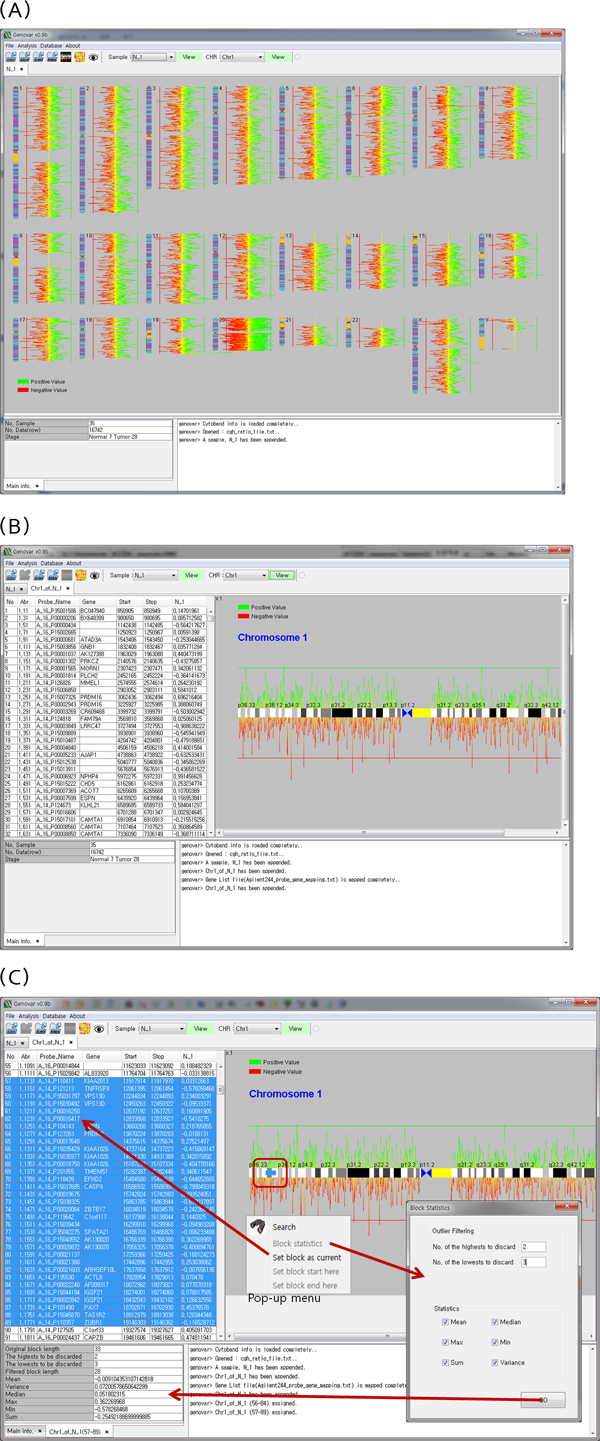
**Chromosomal view and statistical summary**. A. Whole-chromosome scale view of the first sample in the file. Duplication and deletion regions are represented by green and red color, respectively. B. A series of log ratio values for a specific chromosome are given in table form, and a plot of each log ratio value is also offered, along with a cytoband. Zoom in and out functions are also included, via a pop-up menu. C. Statistical summary including mean, median, max, min, sum, and variance, as well as an outlier filter, for a particular region, in single-chromosome view.

After loading an aCGH input file, Genovar displays a view of the aCGH value corresponding to the first sample in the file in a whole-chromosome context (Figure 2A). Users can choose other samples from a toolbar menu. In the resulting view, duplication and deletion regions are represented by green and red colors, respectively. For further analysis, Genovar also provides a detailed view on the single-chromosome scale, with log ratio values related to a specific chromosome given in table form. A plot of each log ratio value is also offered, along with a cytoband view, including zoom in and out functions (Figure 2B). Log ratio values, in table format, are automatically scrolled by clicking on a specific position of the cytoband view. If data on mapping between gene and probe are loaded as well, then a gene name for each record is provided instead of just a chromosome number.

### Elimination of spurious signals

As mentioned before, due to limitations such as low resolution of array platforms, platform specificity, and the type of CNV class, current CNV analysis tools often produce false positive results. For the CNV detection process, the segmental mean of log2 value of probes in the CNV region is the most important value for defining the CNV region. Moreover, noisy log2 values of probes in a CNV region may hinder the discrimination of a spurious signal from a true signal. Therefore, it is necessary to manually separate spurious signals from true signals by visual inspection.

To this purpose, Genovar provides a statistical summary pertaining to the region of interest (Figure 2C) given in single-chromosome view using a pop-up menu. This statistical summary includes mean, median, max, min, sum, and variance, as well as an outlier filter, for a particular region; user-defined high or low values are discarded as outliers. Genovar's visual inspection function was adopted for our CNV detection analysis [26].

### Copy number detection and reporting known CNV region

**Figure 3:**
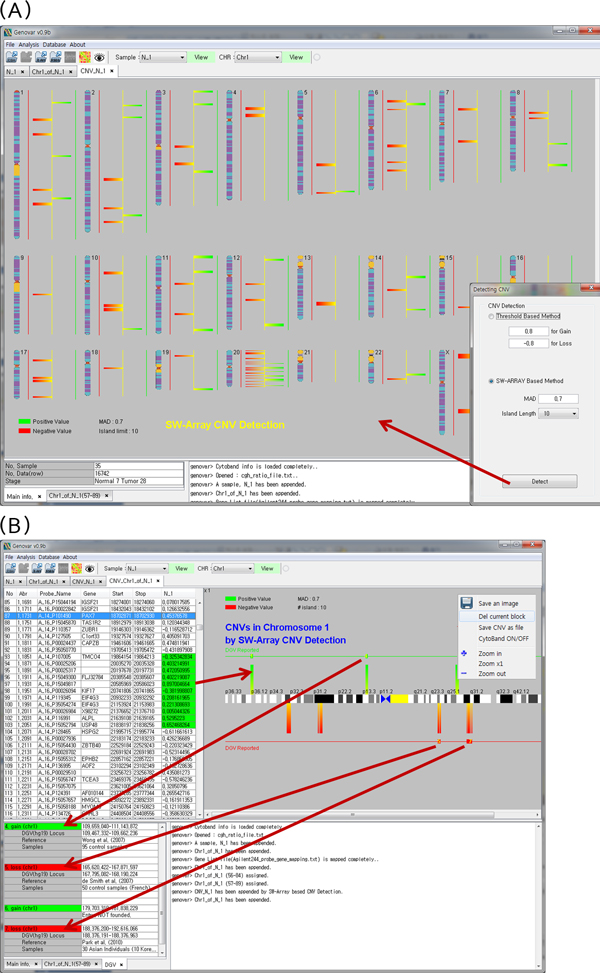
**Copy number variation (CNV) detection**. *A*. Copy number variation (CNV) detection in whole-chromosome view. *B*. Database of Genomic Variants (DGV) entries related to detected copy number variation (CNV) regions. CNV regions reported in DGV are indicated. Gained and lost areas are marked as green and red rectangles, respectively.

Genovar detects copy number variant regions using the Smith-Waterman Array (SW-ARRAY) algorithm [23]. Users input parameters such as median absolute deviation (MAD) and island block length to start the algorithm (Figure 3A). Setting higher MAD value and island block length results in stricter CNV region detection. CNV regions can therefore be analyzed on a whole-chromosome scale, thus allowing the selection of specific chromosome regions (Figure 3B) for further detailed analysis. CNV regions can then be related to entries in the Database of Genomic Variants (DGV, http://projects.tcag.ca/variation) [24]. Figure 3B shows the gained and lost regions marked as small green and red rectangles, respectively. Because most scientists want to verify whether identified CNV regions have been previously reported or not, this is a useful function for the user. Additional information including region boundaries for a given locus, gene name, and references related to a reported region are provided in a window at the bottom of the display. Genovar works with the Global UCSC database on the web to access DGV information.

### Genomic comparison with multiple array CGH samples

**Figure 4:**
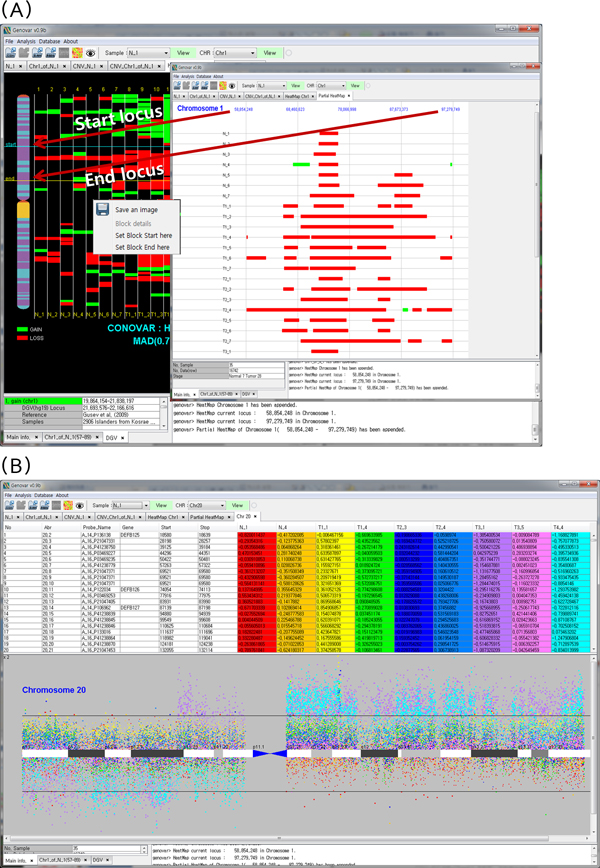
**Comparison with multiple samples**. **A**. Heat map of copy number variation (CNV) regions and detailed view of specific CNV regions. This view is obtained by setting the starting and ending position using a pop-up menu. A heat map is shown for each chromosome. **B**. Comparative genomic hybridization (CGH) values and comparison of samples. Scatter dots represent the log2-scale signal intensity ratio between sample and reference. The colored samples correspond directly to the same colored spot on the cytoband view.

Another useful function of Genovar is the comparison of CNV regions or aCGH values between samples. Genovar displays CNV regions in a specific chromosomal view named heat map (Figure 4A), which enables the user to query details regarding a particular region; detailed CNV regions with absolute loci are obtained by assigning starting and ending positions. Comparison of aCGH log ratio intensities between samples is another common analytical task. To handle this, our system supports the display of aCGH values for a given sample in comparison with those for other samples. In Figure 4B, nine samples chosen by the user are displayed together. Samples are distinguished by columns highlighted by color. Each color directly corresponds to the same colored spot in the cytoband view. Thus, differences between samples are easily shown at a glance.

### Displaying sequence alignments and read information from a BAM file

**Figure 5:**
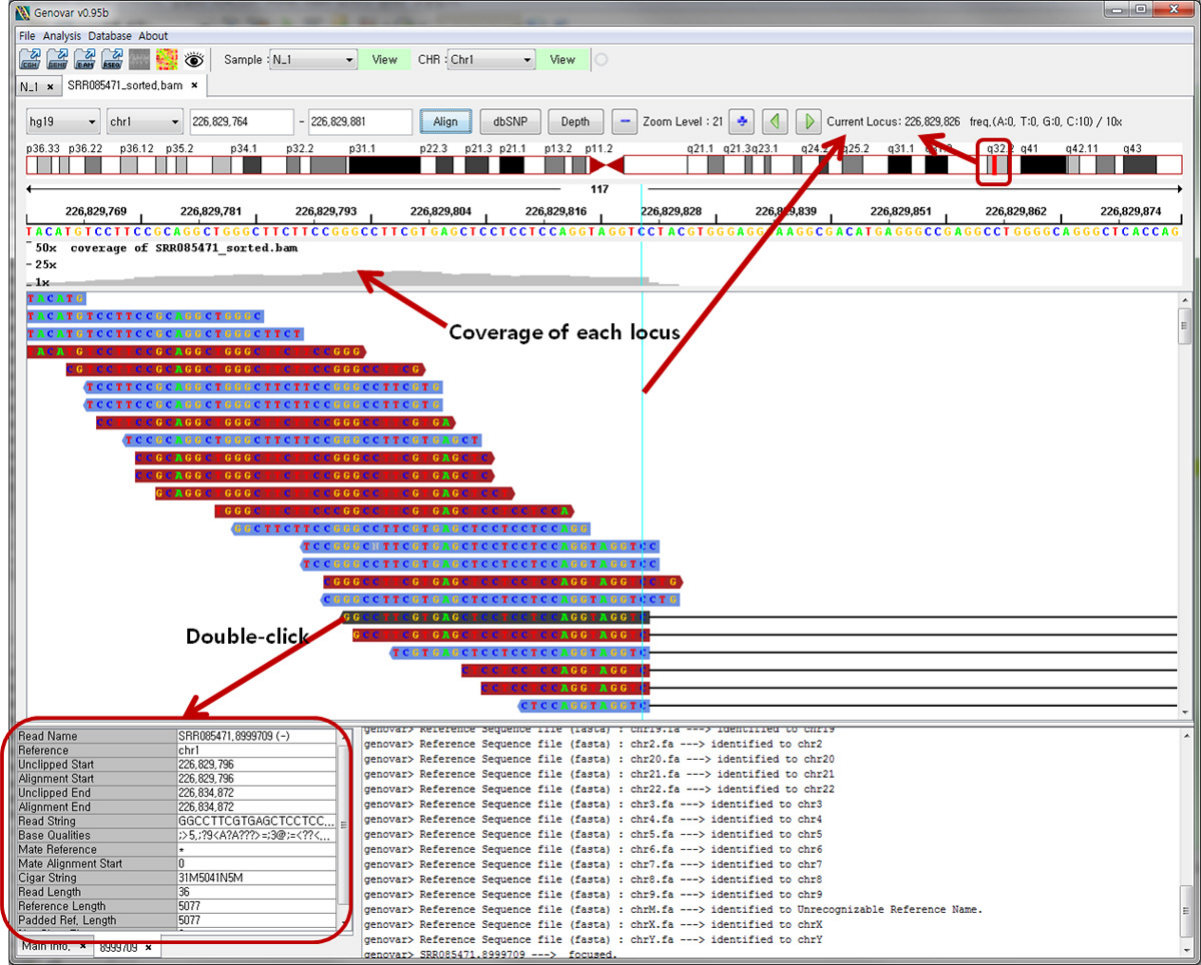
**Sequence alignment view of a single binary sequence alignment/map (BAM) file**. A single alignment view contains cytoband information, locus range, read-depth coverage of each locus, zoom level, and frequency of nucleotides for the current viewing window.

The recent NGS technology allows the discovery of small CNVs. In particular, read-depth coverage of NGS data is a very useful resource to detect homogeneous deletions. Genovar imports BAM formats and displays sequence alignment results. The alignment view (Figure 5) contains cytoband information, locus range, NGS coverage of each locus, zoom level, and frequency of nucleotides in a single viewing window. The user can identify the CNV region using the coverage of each locus on the upper panel. If reference sequence information (e.g., hg19 or hg18, UCSC FastA format) is loaded as well, then each nucleotide base of the corresponding locus is also displayed along with the sample locus.

### Genetic variant inspection based on sequence alignment results

**Figure 6:**
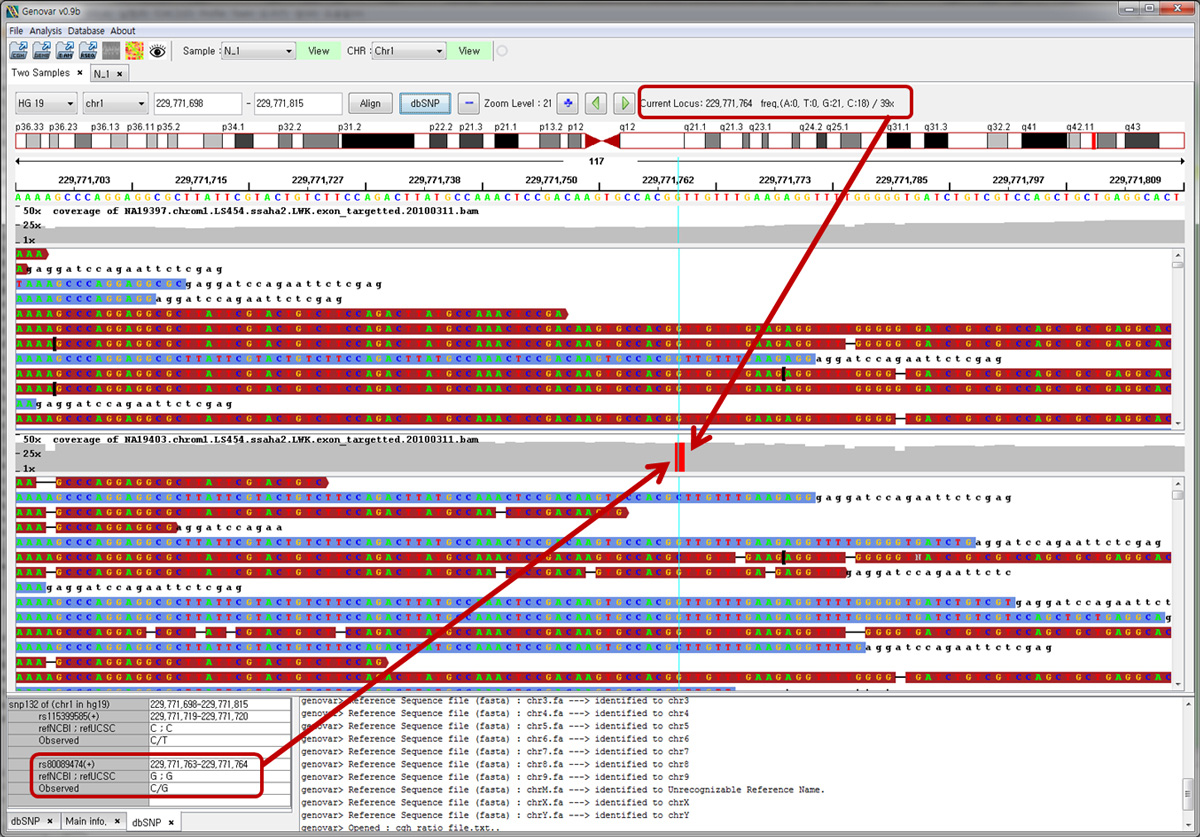
**Sequence alignment comparison of two binary sequence alignment/map (BAM) files**. Comparison of sequence alignment results from one sample with those from another sample. Results from dbSNP in a user-specified locus range are a useful means of detecting unknown SNPs.

Genovar shows multiple sequence alignment results simultaneously (Figure 6). This function is quite powerful because users can differentiate true genetic variants from spurious artifacts based on sequence alignment results. For example, genetic variants such as SNPs, indels, and CNVs derived from external reference databases or various softwares are intuitively accessible in terms of the sequence alignment of the designated genomic region. Calculations of allele frequencies and SNP calling for each sample are performed separately, and differences in SNPs between samples are directly shown. Using this function, the user can find a CNV region containing differences in consecutive SNPs between multiple samples.

## Conclusions

Genovar is a useful tool to detect the CNV with the robust SW-algorithm as a dynamic programming solution and to visualize the detailed CNV information for performing quality control process. The user-friendly graphical interface of Genovar enables the user to identify CNV regions more efficiently. This graphical user interface was implemented by JAVA swing. Moreover, Genovar utilizes two major types of data formats such as those of aCGH or NGS to visualize CNV regions. Genovar compares the detected CNV regions with previously reported CNV regions that have been deposited in DGV/dbSNP. These functions are especially powerful because they allow users to verify whether the CNV regions and SNPs found in their own dataset are truly novel, dramatically reducing time and effort.

Genovar has two distinct advantages over previously reported softwares. It enables users to eliminate spurious ones from true signals through visual inspection and summarized information of detected CNV. Moreover, it even visualizes sequence alignment along with chromosomal regions. For sequence alignment data, Genovar provides a read-depth plot and summarized information of each read when a certain read is selected in the panel.

The visual inspection function of Genovar was used in many practical CNV analysis projects [26]. Via the filtering process, spurious signals were removed. We expect that Genovar enables to analyze CNV more conveniently and accurately via its useful functions.

## Competing interests

The authors declare that they have no competing interests.

## Authors' contributions

KSJ was mainly responsible for the development and set-up of the software, performed the simulations, drafted the manuscript, and wrote most of the methods section. SM and YJK participated in writing the manuscript and designing and testing of the software, and contributed ideas regarding key operations of Genovar; BJK participated in designing and testing of the software. KP revised the manuscript and coordinated the project. All authors read and approved the final manuscript.

## Supplementary Material

Additional file 1 **Genovar user's guide**. This file is the user's guide of Genovar, which was uploaded to the Genovar website http://genovar.sourceforge.net/. Click here for file
