# Supplementary material for: Genovar: a detection and visualization tool for genomic variants
Source: BMC Bioinformatics. 2012 May 8;13(Suppl 7):S12. doi: 10.1186/1471-2105-13-S7-S12 (PMC3348018; doi:10.1186/1471-2105-13-S7-S12)
Supplement: Additional file 1 — Genovar user's guide. This file is the user's guide of Genovar, which was uploaded to the Genovar website http://genovar.sourceforge.net/. [file 1471-2105-13-S7-S12-S1.pdf]

Genovar Quick Guide

Kwang Su Jung (ksjung76@gmail.com)

Division of Bio-Medical informatics

Center for Genome Science

Korea National Institute of Health

1. What is Genovar?

Along with single nucleotide polymorphisms (SNPs), copy number variation (CNV) is considered an important source of genetic variation associated with disease susceptibility. Despite the importance of CNV, the tools currently available for its analysis often produce false positive results due to limitations such as low resolution of array platforms, platform specificity, and the type of CNV. To resolve this problem, spurious signals must be separated from true signals by visual inspection. None of the previously reported CNV analysis tools support this function and the simultaneous visualization of comparative genomic hybridization arrays (aCGH) and sequence alignment. The purpose of the present study was to develop a useful program for the efficient detection and visualization of CNV regions that enables the manual exclusion of erroneous signals.

A JAVA-based stand-alone program called Genovar, which utilizes the Smith-Waterman Array (SW-ARRAY) algorithm to detect CNV regions, was developed. To ascertain whether a detected CNV region is a novel variant, Genovar compares the detected CNV regions with previously reported CNV regions using the Database of Genomic Variants (DGV, http://projects.tcag.ca/variation) and the Single Nucleotide Polymorphism Database (dbSNP). The current version of Genovar is capable of visualizing genomic data from sources such as the aCGH data file and sequence alignment format files.

Genovar is freely accessible and provides a user-friendly graphic user interface (GUI) to facilitate the detection of CNV regions. The program also provides comprehensive information to help in the elimination of spurious signals by visual inspection, making Genovar a valuable tool for reducing false positive CNV results.

Availability: (http://genovar.sourceforge.net/).

2. Main features

The main features of Genovar are summarized below.

- Analyzing/comparing them with multiple aCGH samples and windows.
- Elimination of spurious signals, and statistical block operation of log2 ratio values.
- Identification of CNV regions using the SW-Array algorithm and thresholds.

- Notation of known and unknown CNV in terms of the above discovered CNV regions using DGV.
- Graphic representation of CNV region and aCGH information with multiple samples.
- User-intuitive and fast navigation on chromosomes for retrieving reads in terms of locus range query.
- Graphic-based display of sequence alignment results and read information in BAM (binary sequence alignment/map) format.
- Calculation of read-depth and allele frequency of each locus in the alignment area specified by the user and identification of known SNPs and CNV from dbSNP and DGV, respectively.
- Comparison of sequence alignment results between different samples.

3. System Inputs

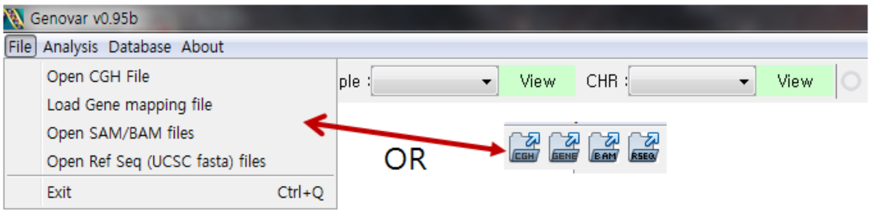

Fig 1. Genovar file input.

The input format of Array CGH contains probe number and name, chromosome number, probe starting and ending locus, and a series of log2 intensity values corresponding to one or more samples. Columns are delimited by ‘TAB’. Array CGH sample formats are available for download on the Genovar website (http://genovar.sourceforge.net/). BAM (binary sequence alignments/Map) are the binary equivalent to SAM (sequence alignments/Map). Genovar uses BAM format to perform sequence-based variation analysis. SAM is a TAB-delimited text format that is easy to parse, generate, and check for errors. However, SAM is somewhat slow to parse, so Genovar imports BAM format files for intensive data processing. Using Samtools software [4], BAM or SAM can be easily converted to the other format, and an index (*.BAI) of the BAM file can also be easily created. BAMs and BAIs should be together in the same directory and have same file name except intension file name. Most sequence-based analysis tools support BAM format. For constructing an index (*.bai), Use samtools with 'index' option.

(Ex) samtools index yourbam.bam youbam.bai

To display reference sequence (hg19/hg18) information, Genovar uses UCSC FastA reference sequence format(*.fa) and its index(*.fai). Reference sequence and its index should be also in the same directory. Users can easily download FastA formats from the UCSC website (http://genome.ucsc.edu/). Other files are downloadable from Genovar web site (http://genovar.sourceforge.net). For constructing a fasta index. Use samtools with 'faidx' option.

(Ex) samtools faidx chr1.fa chr1.fai

4. Chromosomal View from Array CGH Data

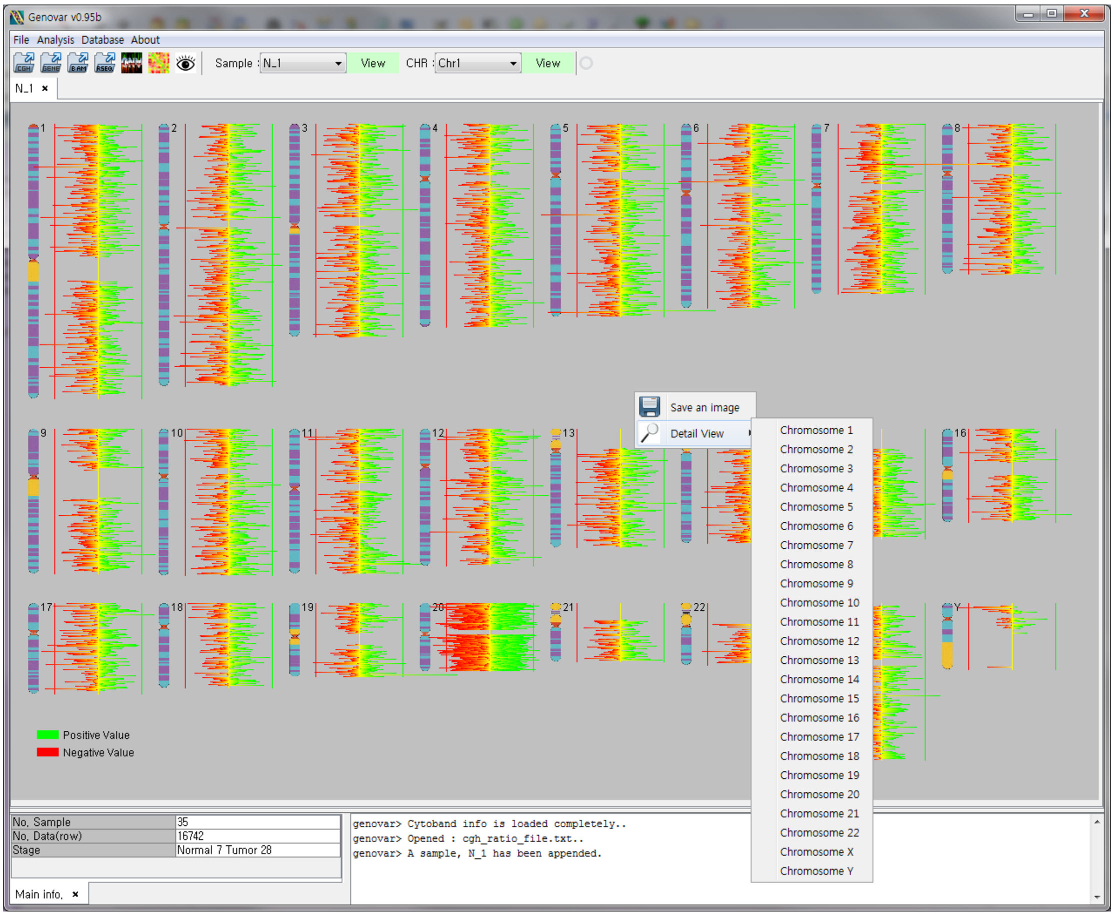

Fig 2. A whole-chromosome view.

After loading an Array CGH input file, Genovar displays a view of CGH value corresponding to the first sample in the file in a whole-chromosome context (Fig 2). Users can choose other samples using a toolbar menu. In the resulting view, hyper- and hypo-expressed regions are represented by green and red color, respectively. For

further, more detailed analysis, Genovar also provides a detailed view on the single-chromosome scale, with log ratio values related to a specific chromosome given in table form. A plot of each log ratio value is also offered, along with a cytoband view, including zoom in and out functions (Fig 2).

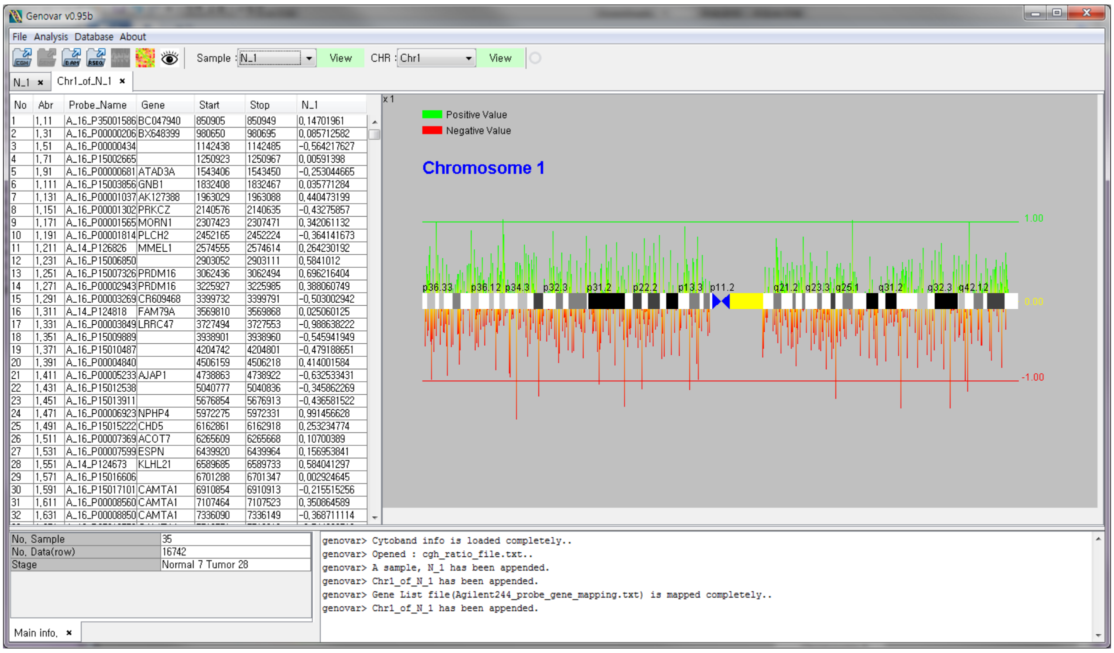

Fig 3. A single-chromosome view.

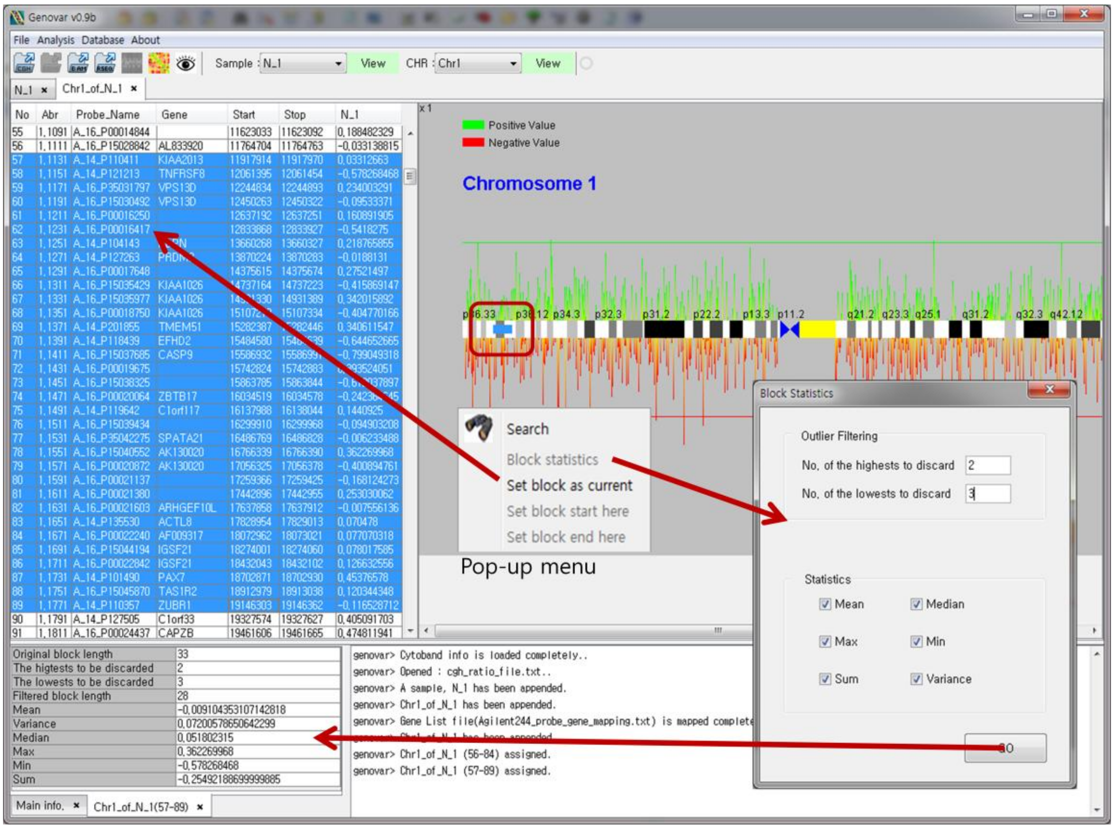

Fig 4. Statistical summary.

Log ratio values, in table format, are automatically scrolled by clicking a specific position of the cytoband view. If mapping information between gene and probe has been loaded as well, a gene name for each record will be given instead of just a chromosome number.

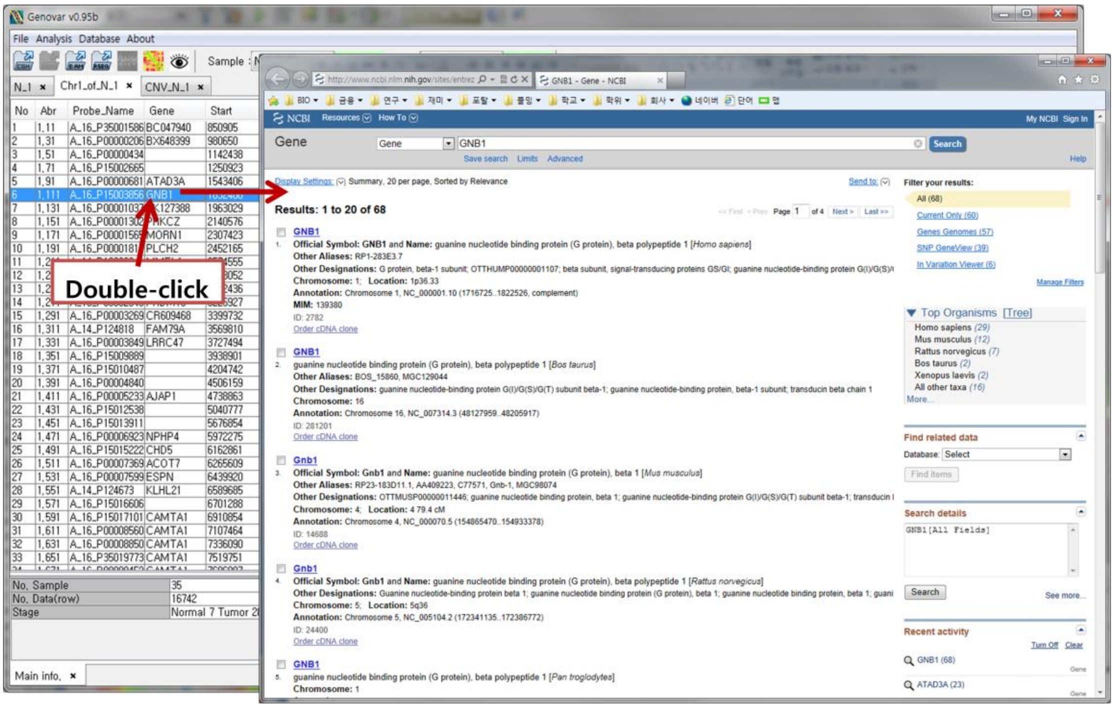

Fig 5. NCBI search.

When user double-clicks a gene name in table, NCBI gene searching is performed if internet connection is available. A statistical summary pertaining to the region of interest (Fig 4) is also given in single-chromosome view using a pop-up menu. This statistical summary includes mean, median, max, min, sum, and variance, as well as an outlier filter, for a particular region; user-defined high or low values are discarded as outliers.

5. Copy Number Detection and Reporting known CNV region.

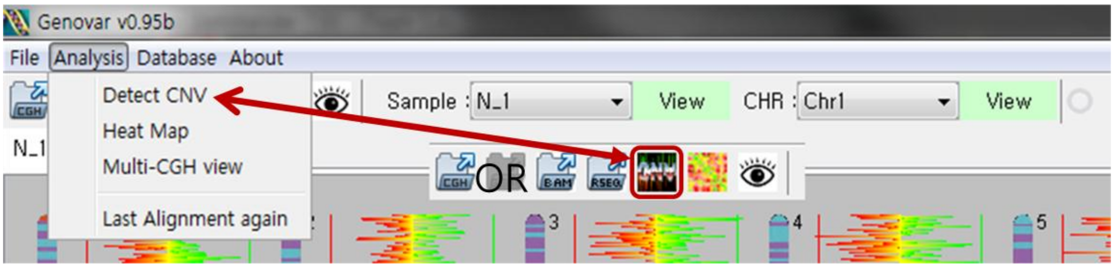

Fig 6. CNV Detection menu.

Genovar detects copy number variant regions using the Smith-Waterman Array (SW-ARRAY) algorithm. Users should input parameters such as median absolute deviation (MAD) and size of island block to start the algorithm (Fig 7). Setting higher MAD values and island block sizes results in stricter CNV region detection.

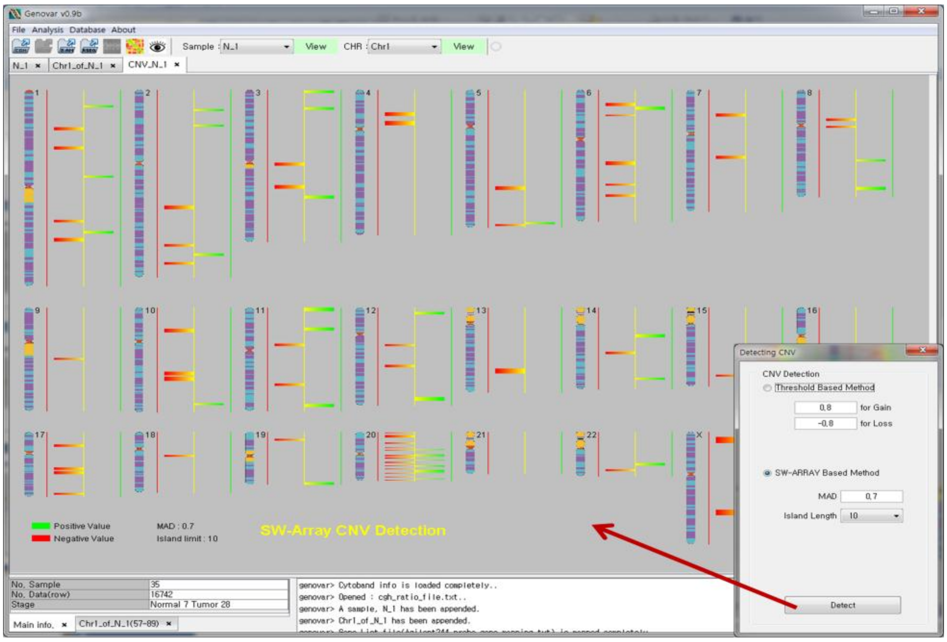

Fig 7. CNV result with SW-Array.

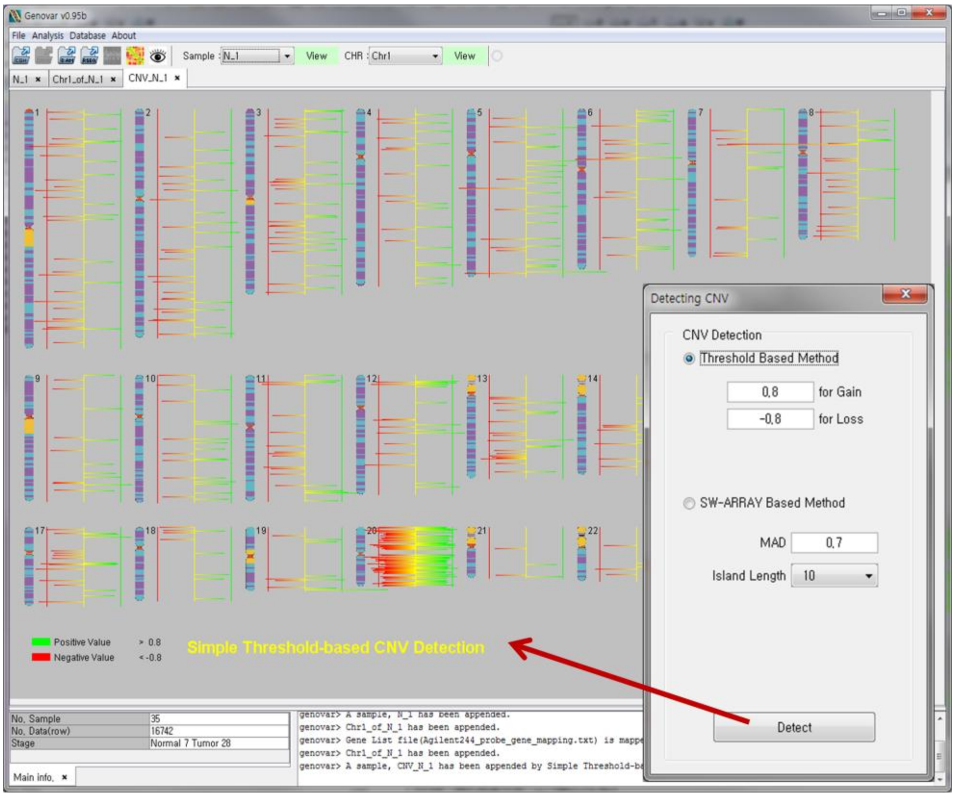

Fig 8. A single-chromosome view with Threshold.

As a result, CNV regions on the whole chromosome scale(Fig 7) are provided, and users can choose specific chromosome regions for further detailed analysis using pop-up menu.

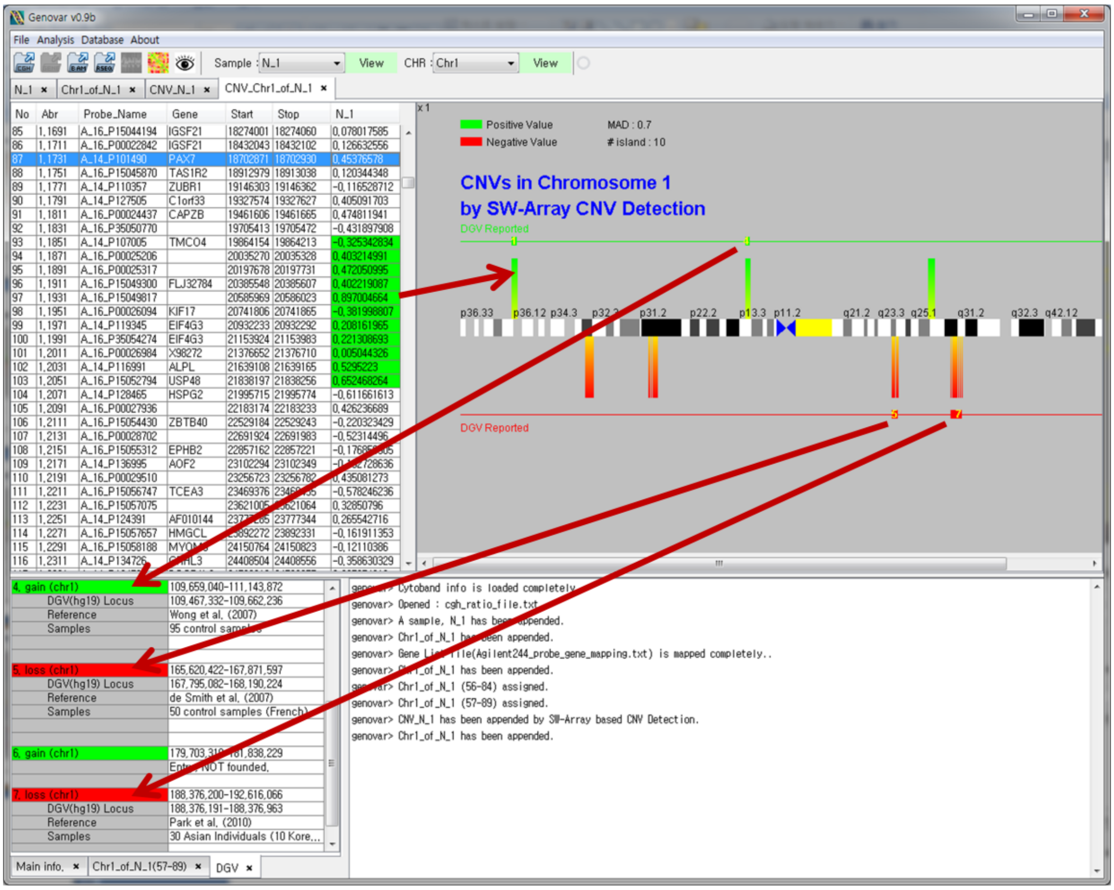

Fig 9. A single-chromosome view of CNV result with SW-Array.

In the CNV region view on the single-chromosome scale (Fig 9), CNV regions reported in the Database of Genomic Variants (DGV, http://projects.tcag.ca/variation) are marked as small green and red rectangles on green and red lines, indicating gained and lost regions, respectively. CNV regions are derived from Fig 7. This might be one of the most useful functions of Genovar, since most scientists want to verify whether or not CNV regions found by themselves have been previously reported. Additional information, such as region boundaries for a given locus, gene name, and references related to a reported region are provided in the table window at the bottom of the display. Genovar works with the Global UCSC database on the web to access DGV information. Thus, an internet connection should be available. DGV distributes sequence information on genomic variants as text files on the DGV website, and these are freely downloadable.

6. Genomic comparison with multiple array CGH samples.

Another useful function of Genovar is comparison of CNV regions (Fig 11) or CGH values between samples (Fig 13).

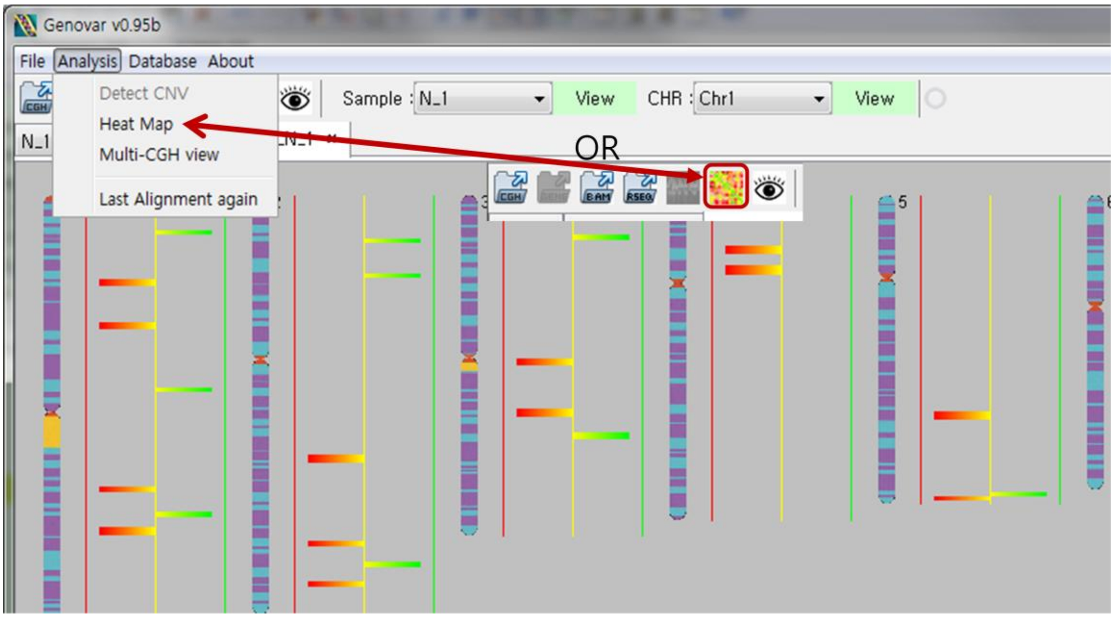

Fig 10. Heat map menu.

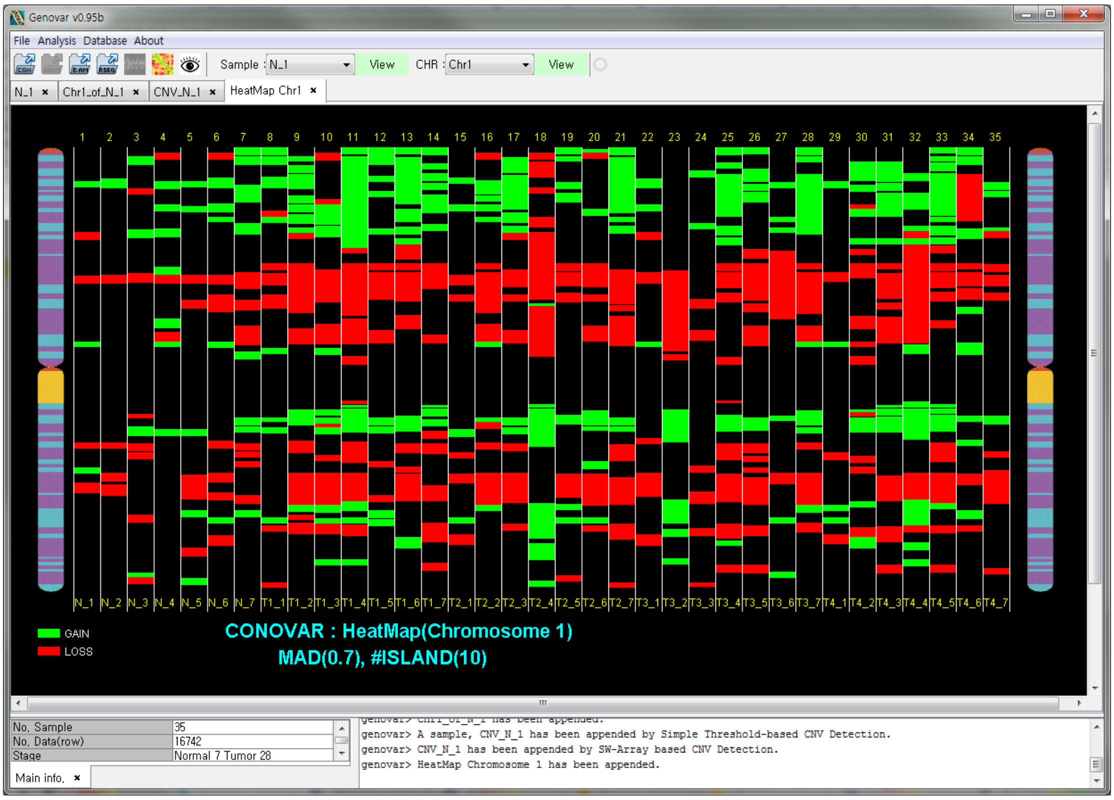

Fig 11. Heat map

Genovar shows CNV regions for all samples in a specific chromosomal view named heat map (Fig 11). In this view, the user can query details regarding a particular region; detailed CNV regions with absolute loci are obtained by assigning starting and ending positions using pop-up menu.

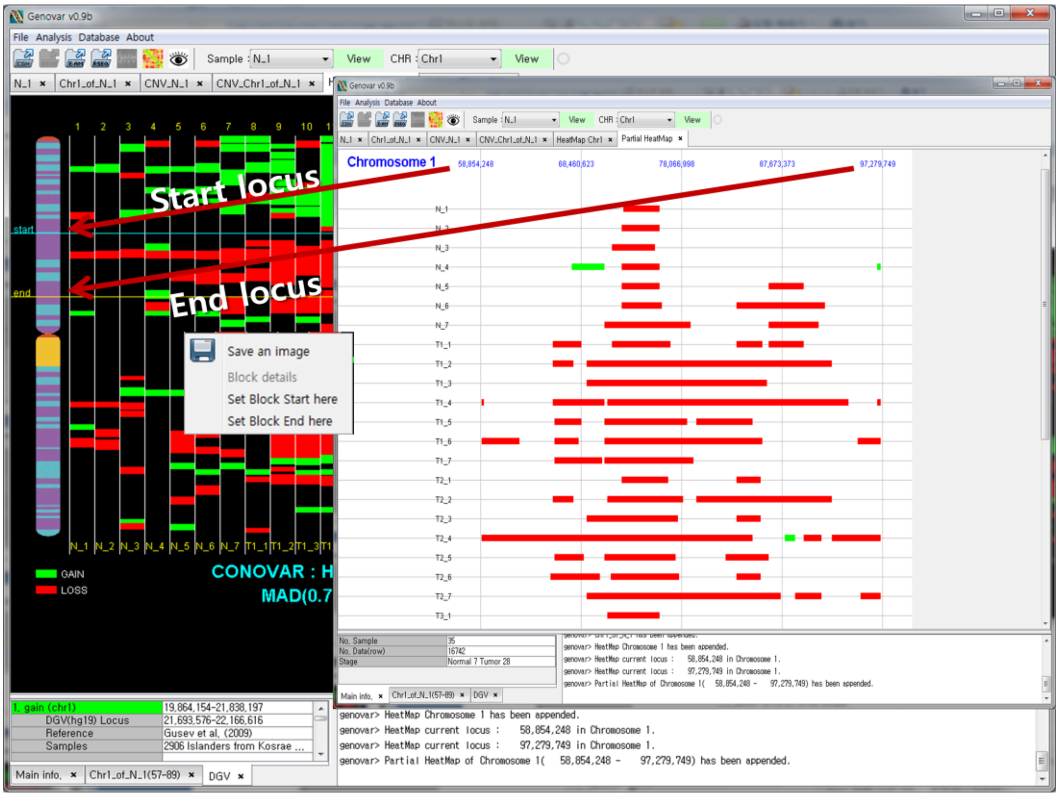

Fig 12. Heat map of specific chromosome.

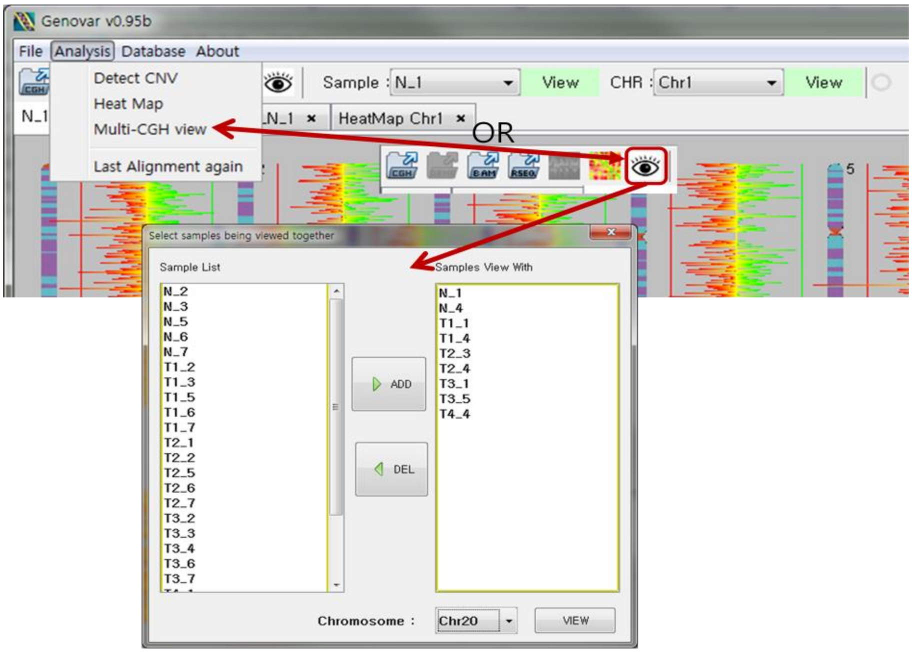

Fig 13. Menu for comparison of samples' CGH values; Multi-CGH view.

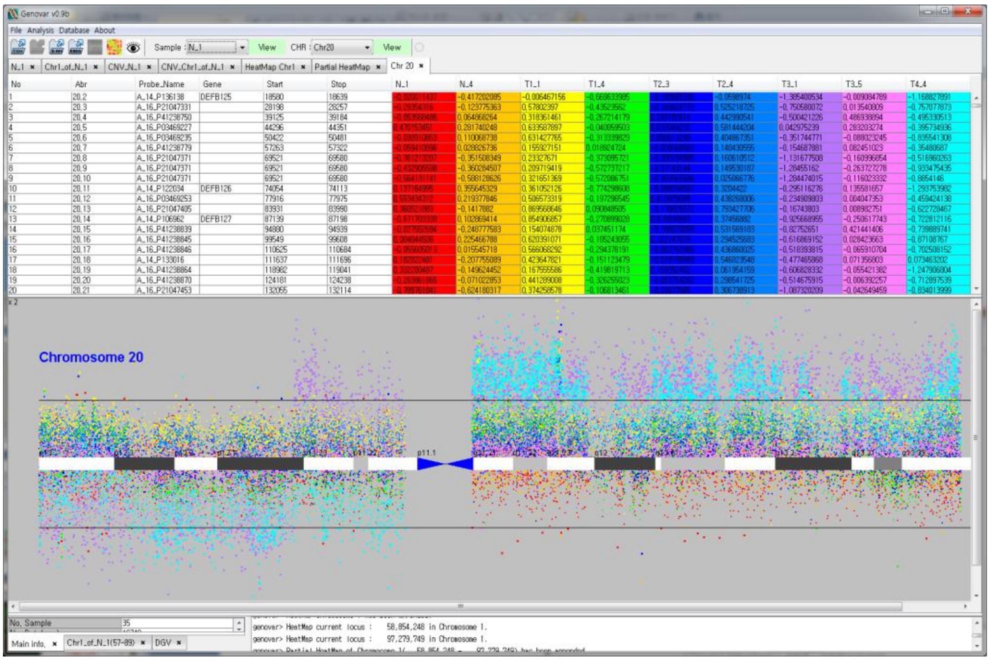

Fig 14. CGH values; comparison of samples.

Comparison of Array CGH log ratio intensities among samples (Fig 14) is another common analysis task. To handle this, our system supports a view of CGH values for a given sample compared with those for other samples. In Fig 14, nine samples chosen by the user are displayed together. Samples are distinguished by columns highlighted by color. Each color directly corresponds to the same colored spot in the cytoband view. Thus, differences among samples are easily shown at a glance.

7. Displaying sequence alignments and read information from a BAM file

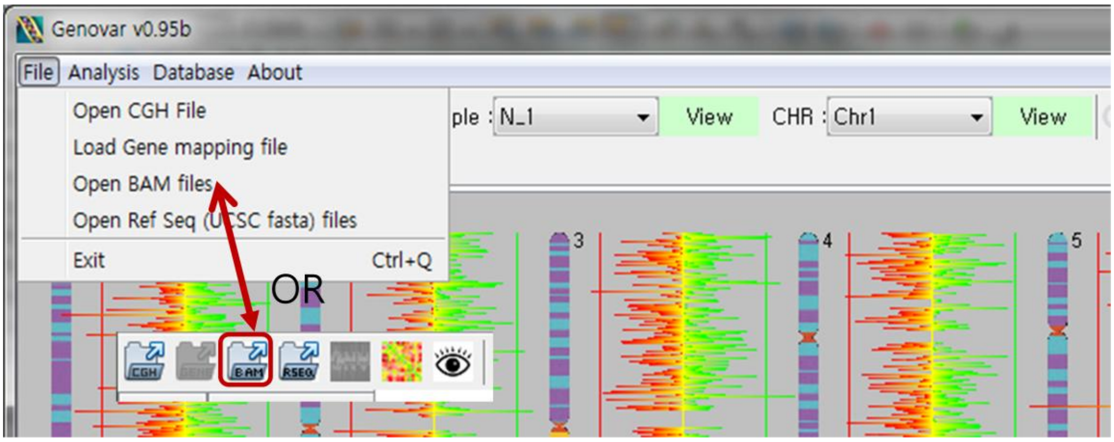

Fig 15. Open bam files.

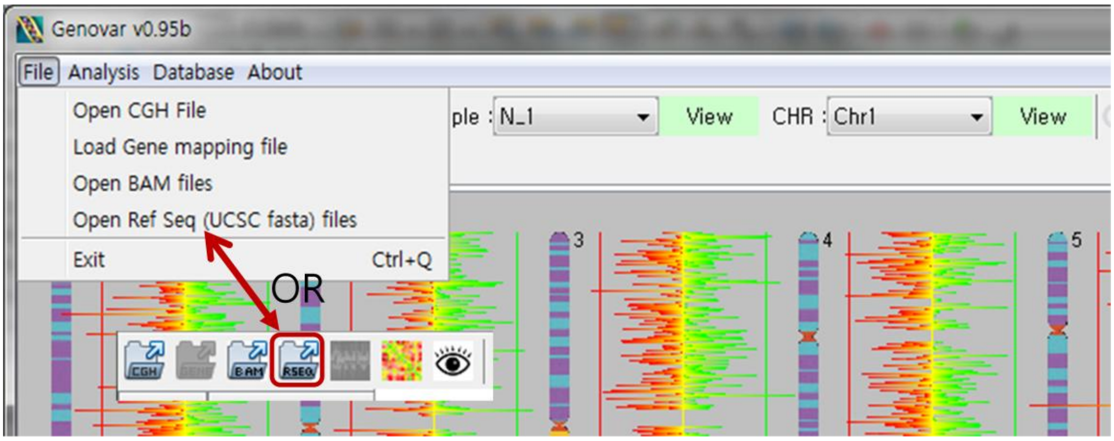

Fig 16. Open reference sequence files (UCSC fasta).

Genovar imports BAM (binary sequence alignment/map) formats to display sequence alignment results. The alignment view (Fig 17) contains cytoband information, locus range, coverage of each locus, zoom level, and frequency of nucleotides in a single viewing window. BAM (binary sequence alignments/Map) are the binary equivalent to SAM (sequence alignments/Map). Genovar uses BAM format to perform sequence-based variation analysis. SAM is a TAB-delimited text format that is easy to parse, generate, and check for errors. However, SAM is somewhat slow to parse, so Genovar imports BAM format files for intensive data processing. Using Samtools software, BAM or SAM can be easily converted to the other format, and an index (*.BAI) of the BAM file can also be easily created. **BAMs and BAIs should be together in the same directory and have same file name except intension file name**. Most sequence-based analysis tools support BAM format. For constructing an index (*.bai), Use samtools with 'index' option.

(Ex) samtools index yourbam.bam youbam.bai

To display reference sequence (hg19/hg18) information, Genovar uses UCSC FastA reference sequence format(*.fa) and its index(*.fai**). Reference sequence and its index should be also in the same directory and have same file name except intension file name**. Users can easily download FastA formats from the UCSC website (http://genome.ucsc.edu/). Other files are downloadable from Genovar web site (http://genovar.sourceforge.net). For constructing a fasta index. Use samtools with 'faidx' option.

(Ex) samtools faidx chr1.fa chr1.fai

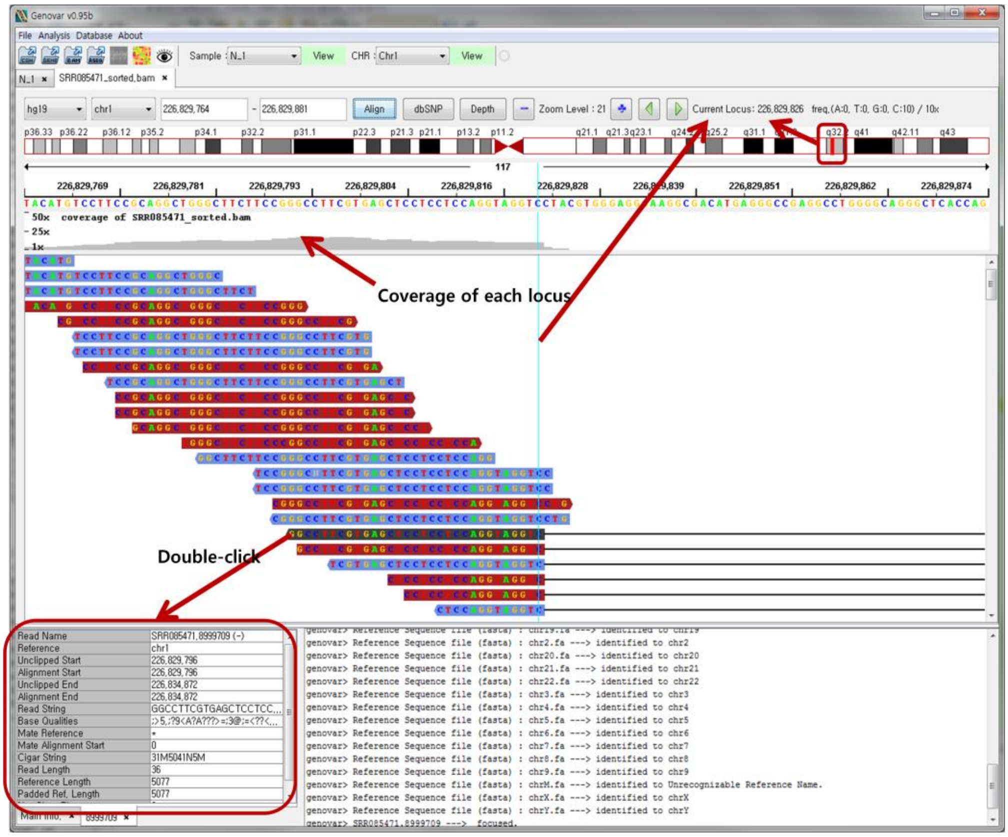

Fig 17. Sequence alignment view of single BAM file.

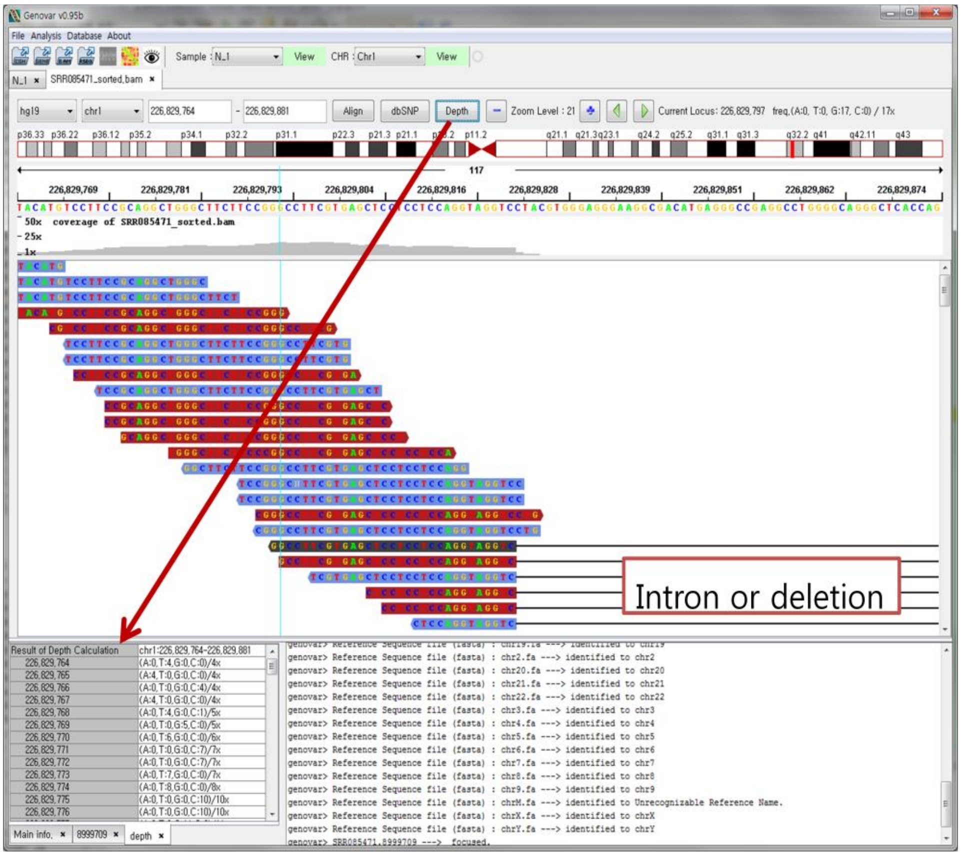

Fig 18. Exporting depth of current window.

If reference sequence information (*e.g.*, hg19 or hg18, UCSC FastA format) has been loaded as well, each nucleotide base of the corresponding locus is also displayed along with the sample locus. Using the mouse, users drag and drop positions of interest on the cytoband and read-alignment panel to navigate a chromosome. Read information is displayed in the table panel at the bottom of the window when the user double-clicks on an interesting read in the alignment panel. At this time, the background color of the double-clicked read changes into dark gray to easily recognize it, even when the window range moves. The red and blue backgrounds of reads (Fig 18) represent forward and reverse directions of mapped reads, respectively. Users can easily download FastA formats from the UCSC website (http://genome.ucsc.edu/) and BAM (and its index) from the 1000 genome ftpsite (ftp://ftp.1000genomes.ebi.ac.uk/vol1/ftp/). Other files including indices of UCSC FastA are downloadable from Genovar web site (http://genovar.sourceforge.net).

8. SNP calling, dbSNP searching and comparing between sequence alignment results

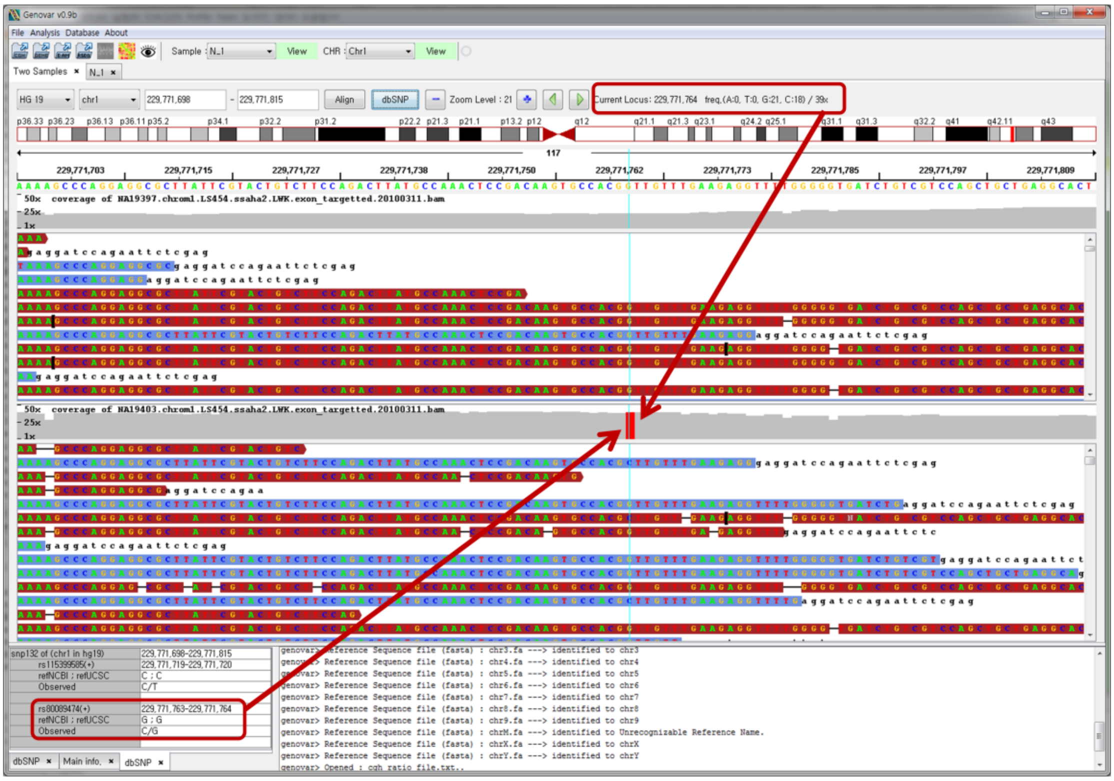

Fig 19. Exporting depth of current window

Genovar shows multiple sequence alignment results simultaneously (Fig 19). This function is quite powerful because differences between two alignment results, for example, in SNP and read mapping information, are intuitively displayed. Calculation of allele frequencies and SNP calling for each sample are separately performed, and differences in SNPs between samples are directly shown. In Fig 19, the user has queried into dbSNP to survey already-reported SNPs, and Genovar has identified known SNPs from dbSNP. In this way, Genovar provides unknown SNP information in particular sequence alignment results to the user. dbSNP versions supported by Genovar include dbSNP132, dbSNP131 of Human Genome 19 (GRCh37), and dbSNP130, dbSNP129, dbSNP128 and dbSNP126 of Human Genome 18 (build 36).

9. Database

Currently, Genovar uses the Database of Genomic Variants (DGV, http://projects.tcag.ca/variation) [1] and Single Nucleotide Polymorphism Database (dbSNP) [2] of the National Center for Biotechnology Information (NCBI) to detect unknown variants. Using Database menu, user can directly connect DGV and dbSNP, and choose version of human genome(hg18/hg19) and dbSNP; dbSNP132, dbSNP131 of Human Genome 19 (GRCh37), and dbSNP130, dbSNP129, dbSNP128 and dbSNP126 of Human Genome 18 (build 36). **Users are required to connect internet for these services**.

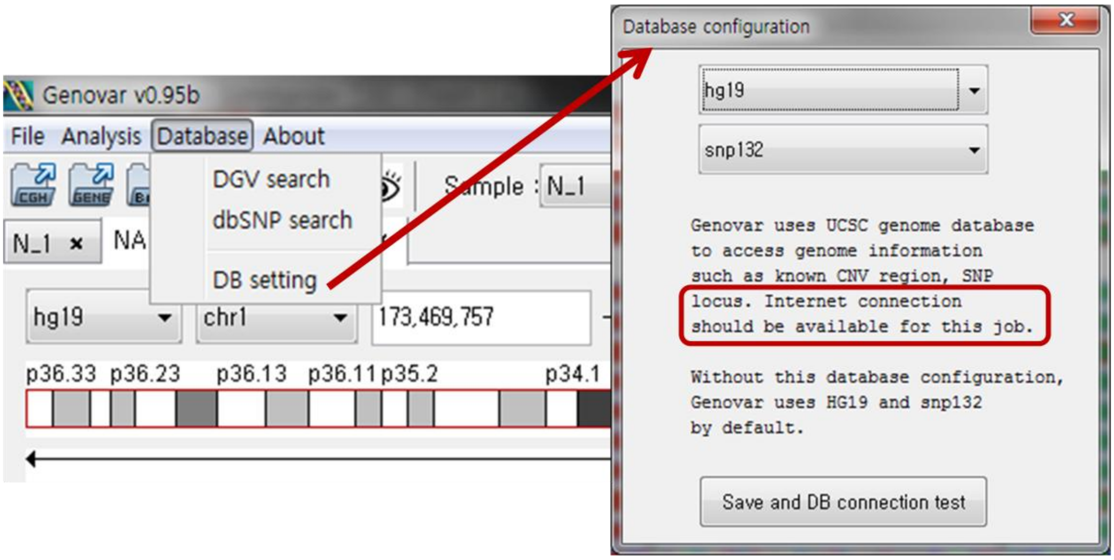

Fig 20. Database setting.

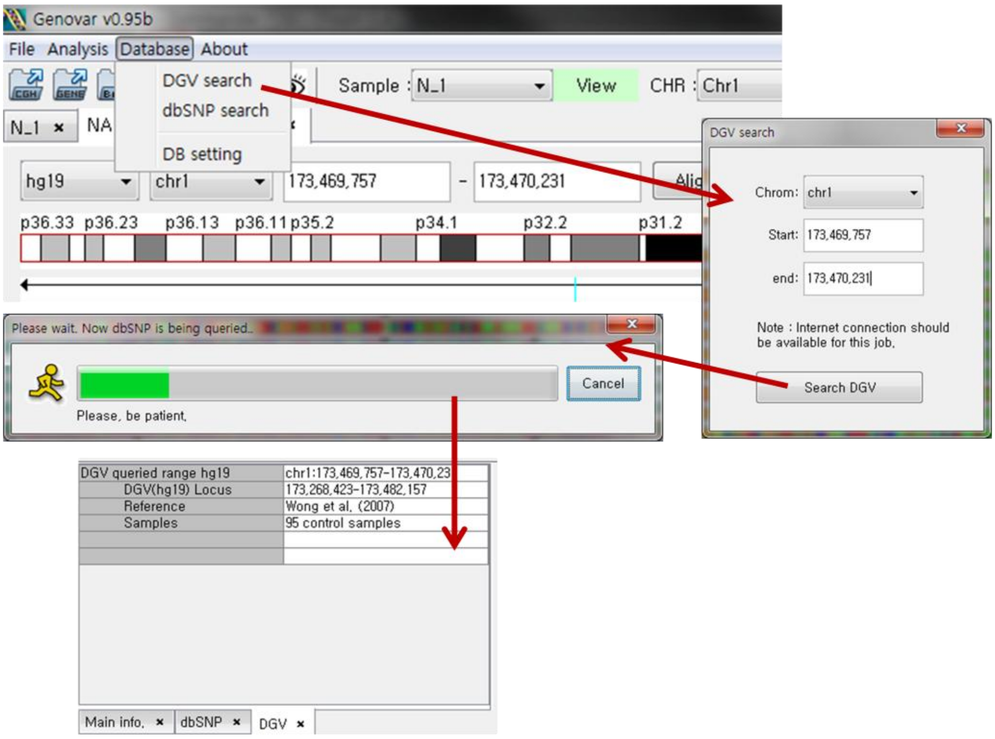

Fig 21. Database; DGV search.

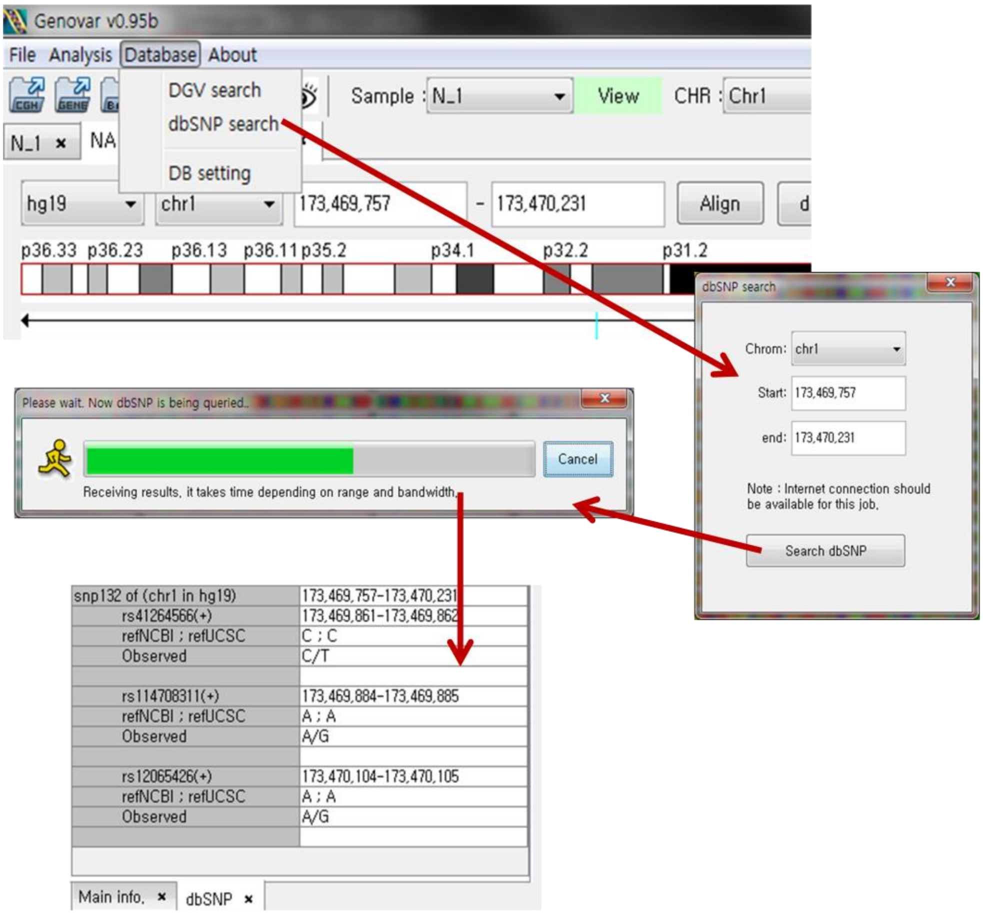

Fig 22. Database; dbSNP search.

Reference

1. Iafrate AJ, Feuk L, Rivera MN, Listewnik ML, Donahoe PK, Qi Y, Scherer SW, Lee C: **Detection of large-scale variation in the human genome**. Nat. Genet. 2004, **36(9):**949-951.
2. Wheeler DL, Barrett T, Benson DA, Bryant SH, Canese K, Chetvernin V, Church DM, DiCuccio M, Edgar R, Federhen S, Geer LY, Kapustin Y, Khovayko O, Landsman D, Lipman DJ, Madden TL, Maglott DR, Ostell J, Miller V, Pruitt KD, Schuler GD, Sequeira E, Sherry ST, Sirotkin K, Souvorov A, Starchenko G, Tatusov RL, Tatusova TA, Wagner L, Yaschenko E: **Database resources of the National Center for Biotechnology Information**. Nucleic Acids Research 2007, **35**:D5–D12.
3. Price TS, Regan R, Mott R, Hedman A, Honey B, et al.: **SW-ARRAY: a dynamic programming solution for the identi-fication of copy-number changes in genomic DNA using array comparative genome hybridization data**. Nucleic Acids Research 2005, **33**:3455–3464.
4. Li H, Handsaker B, Wysoker A, Fennell T, Ruan J, Homer N, Marth G, Abecasis G, Durbin R, 1000 Genome Project Data Processing Subgroup: **The Sequence Alignment/Map format and SAMtools**. Bioinformatics 2009, **25(16)**:2078-2079.
